# 4D-CTA improves diagnostic certainty and accuracy in the detection of proximal intracranial anterior circulation occlusion in acute ischemic stroke

**DOI:** 10.1371/journal.pone.0172356

**Published:** 2017-02-24

**Authors:** Bart A. J. M. Wagemans, Wim H. van Zwam, Patricia J. Nelemans, Robert J. van Oostenbrugge, Alida A. Postma

**Affiliations:** 1 Department of Radiology, Maastricht University Medical Centre, Maastricht, The Netherlands; 2 Department of Clinical Epidemiology, Maastricht University Medical Centre, Maastricht, The Netherlands; 3 Department of Neurology, Maastricht University Medical Centre, Maastricht, The Netherlands; Universitatsklinikum Freiburg, GERMANY

## Abstract

**Introduction:**

In acute ischemic stroke, imaging of the cranio-cervical vessels is essential for intra-arterial treatment selection. Fast, reliable and easy accessible imaging is necessary 24 hours a day, 7 days a week. Radiologists in training and non-expert readers often perform initial reviewing. In this pilot study, the potential benefit of adding 4Dimensional-CT Angiography (4D-CTA) to the patient selection protocol for intra-arterial therapy is investigated.

**Materials and methods:**

Twenty-five datasets of prospectively recruited patients, eligible for intra-arterial treatment, were enrolled. Four radiologists-in-training consecutively reviewed CTA, CT-Perfusion and 4D-CTA (post-processed from CTP datasets) and scored: occlusion-presence and diagnostic certainty (scale 1–10). Time-to-diagnosis was registered.

**Results:**

Arterial occlusion was present in 8 patients. Accuracy improved from 88–92% after CTA and CTP assessment to 96–100% after 4D-CTA assessment (P-values >0,05). Mean diagnostic certainty improved from 7,2–8,6 to 8,8–9,3 (P-values all < 0,05). Mean time to diagnosis increased from 3, 5, 5 and 4 minutes after CTA to 9, 14, 12, and 10 minutes after 4D-CTA.

**Conclusion:**

4D-CTA as an additive to regular CTA and CT-Perfusion in patients with acute ischemic stroke eligible for intra-arterial treatment shows a tendency to increase diagnostic accuracy and improves diagnostic certainty, when reviewed by radiologist in training, while only mildly prolonging time to diagnosis.

## Introduction

In the Western world stroke is one of the leading causes of mortality and disability. [1] With large demographical changes and consequently increased incidence and prevalence ahead, it will remain so for the coming decades. [2]

In acute ischemic stroke, imaging of the brain and cranio-cervical vessels is essential for treatment decision making. [3] Following the adagium “time is brain”, fast, reliable and easy accessible imaging is necessary 24 hours a day, 7 days a week.

Imaging allows for differentiation between hemorrhagic and ischemic stroke, reveals extend of the ischemia and allows reliable patient selection for intra-arterial therapy (IAT) treatment (clot formation and location, stenosis and infarct-area). [3] As IAT recently has been shown to be beneficial in acute ischemic stroke caused by proximal intracranial anterior circulation occlusion, the role of imaging is even more essential. [4–8]

Patient imaging and selection in acute ischemic stroke can be done with CT Angiography (CTA) alone, or with additional CT-Perfusion (CTP). Imaging assessment is largely done outside regular office hours, under great time pressure and by radiologists in training or non-specialized radiologists on call, making assessment more prone to misdiagnosis with sustained consequences throughout the patient’s further treatment.

A relative new technique for the assessment of intra-cerebral artery occlusion is 4Dimensional CTA (4D-CTA). Recent publications showed that 4D-CTA (also known as time-resolved CTA) is better in defining clot burden and collateral score than conventional CTA in anterior circulation stroke. [9–11] Similar results can be seen when 4D-CTA images are derived from CTP data, a technique in which 4D-CTA is constructed by extra post-processing from the already acquired perfusion data set. Hereby not adding to the imaging protocol, only extending the post-processing protocol. This preventing additional contrast injection and radiation dose, as is the case in dedicated 4D-CTA scans. [12]

The main objective of our study is to asses the benefits of 4D-CTA as additional tool to regular CTA and CTP in the diagnostic performance and certainty of non-expert radiologists (-in-training).

## Materials and methods

### Study design

Twenty-five datasets of prospectively recruited patients, who were clinically eligible for IAT, were analyzed. Patients were consecutively included from July to October 2014. Eligibility for inclusion comprised of a clinical diagnosis of acute stroke, with a deficit on the NIH stroke scale of 2 points or more, intracranial hemorrhage ruled out by non-contrast enhanced CT (NECT), possibility to start IAT within 6 hours from onset, and age 18 or over. [4]

The Maastricht University Medical Centre ethical committee approved the research protocol. (METC 15-4-037). Informed consent for this specific study was waived by the ethical committee.

### Image acquisition and reconstruction parameters

All CT-examinations were performed on a dual source 128-slice CT scanner (SOMATOM Definition Flash, Siemens Healthcare, Germany). Scan protocol consisted of a NECT followed by CTP and arterial phase Dual Energy-CTA (DE-CTA). NECT was acquired in cranio-caudal direction at 120 kVp, 310 mAs, slice-thickness 1 mm and acquisition 128*0,6. CT-Perfusion was acquired starting at the external auditory canal with a 100 mm coverage of the brain, at 80 kVp, 100 mAs, acquisition 32*1,2; rotation time 0,28 s, slice thickness of 5 mm for calculation of perfusion maps; 5 mm reconstructions were used for CTP and 1 mm reconstructions were used for 4D-CTA. Contrast dose for CTP was 50 ml (Ultravist 300 mg/ml, Bayer, Germany) and 40 ml saline, injected trough an 18 Gauge cannula with an injection rate of 7 ml/s using a double piston injector (Medrad, Bayer, Germany). Scan beginning was timed at 2 sec after injection. Arterial phase DE-CTA was performed in caudo-cranial direction, starting at the level of the aortic arch until the pericallosal artery, with a slice thickness of 1mm, tube A 80 kVp, 392 mAs, tube B 140 kV 196 mAs; acquisition 32 * 0,6; collimation 0,6 and rotation time 0,5 sec. Reconstruction parameters for CT-Perfusion and DE- CTA were largely the same (reconstruction kernel H20f, increment 5 mm) except for slice thickness (5mm and 1mm for CTP and 1 mm for DE-CTA). Contrast dose for DE-CTA was 95 ml of contrast (Ultravist 300 mg/ml, Bayer, Germany) and 40 ml of saline, with an injection rate of 7 ml/s. Contrast bolus triggering was performed at the ascending aorta, starting with a delay of 2 seconds after reaching the Hounsfield Unit (HU) threshold of 250.

### Image analysis

**Fig 1:**
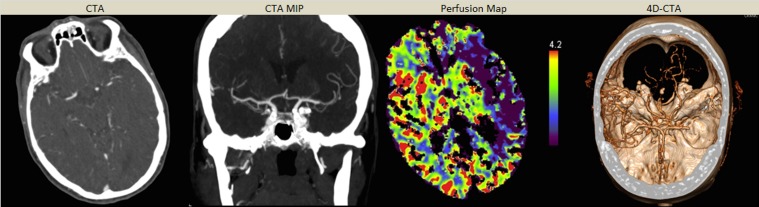
Reviewing example of a patient that presented to the ER with right-sided hemiplegia and dysarthria. A difficult to read CTA due venous enhancement just siding the right MCA was performed, and intra-cranial vessels where initially declared patent. Only when reviewing the 4D-CTA data, the patient was transferred to the angio-suite for IA therapy. Below figs shows consecutively CTA, coronal MIP view, perfusion map and a freeze of the 4D-CTA.

Data sets were anonymized and loaded subsequently into a dedicated workstation (Syngovia MMVP, Siemens Healthcare, Forchheim Germany), which is used in daily practice for image evaluation in stroke patients. Data sets were stripped from patient characteristics and clinical information. Datasets were reviewed by 4 radiologists in training in different stages of training (first to last year radiologists in training). Reviewers were provided with the clinical data that had been available at patient presentation in the original emergency setting, but were blinded to the clinical outcome and radiology report. Reviewers had to consecutively review initial CTA alone, CTA and CTP and finally CT, CTP and 4D-CTA. All post-processing including, CTA, MIP, 3D, CTP and 4D-CTA, could be reconstructed using the Syngovia software, and was allowed at personal preference. Each reviewer performed the post-processing of the CTA and the calculation of the perfusion maps individually. Subsequently they performed 4D-CTA post-processing from the initial perfusion data set and could review it at their own personal preference. (Fig 1). The 4D-CTA was calculated from the 1mm CTP dataset with a standard automated package of MMWP “Dynamic Angio” with preselected programmed default parameters. All interpretation times were recorded (in seconds/minutes). Timing started at the moment of opening the CTA and ending after definitive diagnosis was given after reviewing of the 4D-CTA. Interpretation time includes both reviewing and post-processing time.

The location, if present, and the diagnostic certainty of the findings (scale from 1–10, 10 being absolutely certain about their findings) were noted. Reference standard was defined as consensus reading between two expert (diagnostic and interventional) neuro-radiologists (AAP, 13 years of experience and WVZ 18 years of experience), which reviewed all imaging at their disposition, including CTP, CTA, 4D-CTA and if performed DSA.

### Statistical analysis

Sensitivity, specificity and accuracy were computed for each individual reader from 4x4 contingency tables. These estimates were compared between CTA alone, CTA plus CTP and CTA plus CTP plus 4D CTA by using the McNemar test for paired proportions. Receiver operating characteristic (ROC) curves were constructed to visualize the increase in diagnostic performance after adding CTP to CTA and adding 4D CTA to CTA + CTP. Increase in areas under the curve (AUC) was tested for statistical significance by a non-parametric test for comparing the areas under two or more correlated ROC curves. [13]

Mean values for diagnostic certainty and interpretation time were compared using a t-test for paired samples. P-values ≤0.05 were considered to indicate statistical significance.

Analyses were performed with Statistical Package for the Social Sciences (version 25; IBM, Armonk, New York, USA) and STATA (version 12; StataCorp LP, College Station, TX, USA).

## Results

Eight patients had a proximal intra-cranial artery occlusion. Table 1 shows the locations of the occlusions. Most of the occlusions were in the proximal MCA MI segment (5/8). Other locations were in the MII segment (2/8) and in one case the occlusion was in the internal carotid artery. The 4 readers missed a total of 4 MII and 2 MI occlusions on CTA alone. After 4D-CTA they missed a total of 2 MII occlusions and no MI occlusions.

**Table 1:** Location of the 8 occlusions.

No.	Location
1	Right MCA MII
2	Left MCA MI
3	Left MCA MI
4	Left carotid and MI
5	Right carotid
6	Right MCA MI
7	Right MCA MII
8	Left MCA MI

Reader 1, being the most experienced, proved to be the best performing reader in regards of sensitivity (100%), with slightly lower specificity (94%). Reader 4, being the least experienced reader, had higher specificity (100%) with a slightly lower sensitivity (88%). All results are presented in Table 2. In 4 out of 4 readers there was an increase in overall sensitivity, specificity and consequently accuracy if CTP and 4D-CTA were added to the CTA. There was a slight decrease in specificity in readers 2 and 3 when comparing CTA + CTP to CTA alone. Only reader 3 improved to 100% accuracy after the 4D-CTA (Table 2). No increase in sensitivity or specificity was significant in any of the readers using the McNemar test, *P =* >0,05.

**Table 2:** Performance of the individual readers 1 to 4. Sensitivity, Specificity and accuracy in %. Diagnostic Certainty Scale 1–10 (10 being absolutely certain). Mean interpretation time in sec.

	Sensitivity (95% CI)	Specificity (95% CI)	Accuracy (95% CI)	Diagnostic Certainty mean (+- SD)	Interpretation Time mean (+-SD)
Reader 1					
CTA	100% (8/8) (63% - 100%)	88% (15/17) (64%-99%)	92% (23/25) (64-99%)	8,6 (0,81)	200 (50)
CTA/CT-P	100% (8/8) (63% - 100%)	88% (15/17) (64%-99%)	92% (23/25) (64-99%)	8,8 (0,66) *P*= 0,185	359 (83) *P*=0,000
CTA/CTP/4D-CTA	100% (8/8) (63% - 100%)	94% (16/17) (71%-100%)	96% (24/25) (69-100%)	9,3 (0,59) *P*= 0,001	528 (99) *P*=0,000
Reader 2					
CTA	63% (5/8) (24%-91%)	100% (17/17) (80%-100%)	88% (22/25) (64%-97%)	7,4 (0,87)	321 (144)
CTA/CT-P	75% (6/8) (35%-97%)	94% (16/17) (71%-100%)	88% (22/25) (61%-99%)	8,6 (1,12) *P*=0,000	553 (171) *P*=0,001
CTA/CTP/4D-CTA	88% (7/8) (47%-100)	100% (17/17) (80%-100%)	96% (24/25) (70%-100%)	9,5 (0,87) *P*=0,000	848 (207) *P*=0,000
Reader 3					
CTA	88% (7/8) (47%-100)	94% (16/17) (71%-100%)	92% (23/25) (64%-100%)	7,2 (0,72)	315 (126)
CTA/CT-P	88% (7/8) (47%-100)	88% (15/17) (64%-99%)	88% (22/25) (59%-99%)	7,7 (0,85) *P*= 0,005	580 (202) *P*=0,001
CTA/CTP/4D-CTA	100% (8/8) (63% - 100%)	100% (17/17) (80%-100%)	100% (25/25) (75%-100%)	8,6 (0,91) *P*= 0,000	740 (219) *P*=0,000
Reader 4					
CTA	75% (6/8) (35%-97%)	100% (17/17) (80%-100%)	92% (23/25) (67%-99%)	6,7 (1,33)	237 (71)
CTA/CT-P	75% (6/8) (35%-97%)	100% (17/17) (80%-100%)	92% (23/25) (67%-99%)	7,4 (1,35) *P*= 0,022	406 (96) *P*=0,000
CTA/CTP/4D-CTA	88% (7/8) (47%-100)	100% (17/17) (80%-100%)	96% (25/25) (70%-100%)	8,6 (0,87) *P*= 0,000	563 (111) *P*=0,000

**Fig 2:**
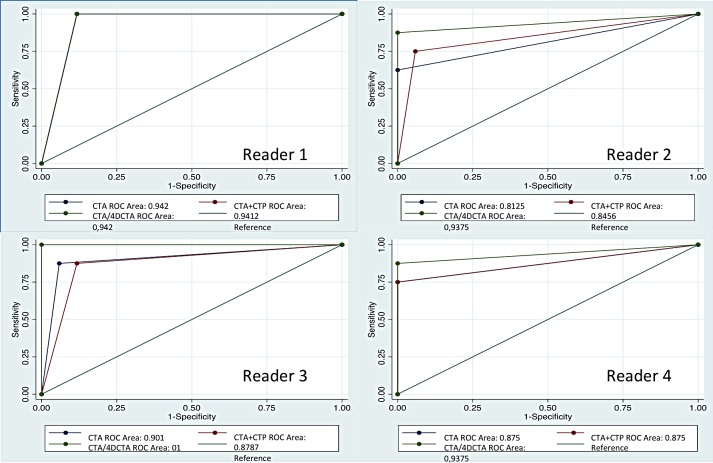
ROC curves and area under the curve (AUC) for diagnostic performance. Four different readers, comparing CTA alone, CTA + CTP, and CTA, CTP and 4DCTA.

To illustrate accuracy reader-operator characteristic (ROC) curves are calculated for all four reviewers (Fig 2). The graphs clearly indicate an increase in the area under the curve (AUC) when 4D-CTA is added but the increase was not significant using the roccomp command in STATA *P* = >0,05.

**Fig 3:**
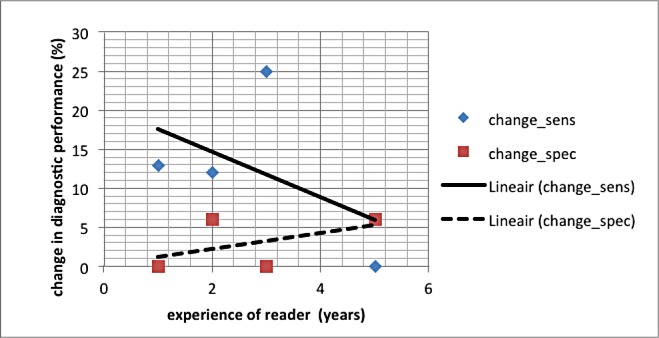
Changes in sensitivity and specificity in relation to the experience of the different readers. Sensitivity and specificity in %, experience in years.

Sensitivity increase after adding 4D-CTA show a trend to be larger in the less experienced readers than in the more experienced readers. Changes in specificity were minimal. (Fig 3)

Diagnostic certainty improved in all readers when 4D-CTA was added. *P*-values for the increase in diagnostic certainty were significant (<0,05) in all readers (Table 2). The least experienced readers (reader 2, 3 and 4) had a lower mean diagnostic certainty of 7.4, 7.2 and 6.7 versus the most experienced reader (reader 1) 8.6 after the initial CTA. This improved after CTP and 4D-CTA to 9.3 for reader 1, 9.5 for reader 2 and 8.6 for readers 3 and 4. Additional 4D-CTA and CTP showed the most benefit for the least experienced readers in term of diagnostic certainty, and a small benefit for the more experienced radiologist in training.

Time to diagnosis, which was timed in all readers, varied widely between the different readers. Time to diagnosis after the CTA varied from 3 to 5 minutes, while time-to-diagnosis after CTP varied from 6 to 10 minutes, and varied from 9 to 14 minutes after 4D-CTA. All increases in time-to-diagnosis were significant *P* = <0,05. There seems to be no relation in time-to-diagnosis and experience of the readers, as the most experienced and the least experienced were the fastest, while reader 2 and 3 demonstrated longer assessment times.

## Discussion

Our study aimed to determine the diagnostic value of 4D-CTA as an additive to the post processing protocol of regular CTA and CTP in the evaluation of acute ischemic stroke patients eligible for intra-arterial treatment in a situation similar to clinical practice. Deliberately choosing non-expert readers: radiologists in training, who usually assess these images first when patients arrive outside office hours. Time to diagnosis and diagnostic certainty of the readers are important parameters when decisions have to be made under time pressure. Previous studies have demonstrated improvement of sensitivity and diagnostic certainty when adding CTP. [12] We demonstrate an additional increase after adding 4D-CTA to the post-processing protocol.

The performance in detecting proximal artery occlusion of the anterior circulation shows a tendency to improve in 3 out of 4 readers when 4D-CTA is added to the regular CTA and CTP. The performance shows a tendency to improve in all 4 four readers, as demonstrated with the AUC of the ROC curves (Fig 2). This indicates that adding 4D-CTA to the imaging protocol in the assessment of ischemic stroke patients eligible of intra-arterial treatment might be beneficial for the performance when reviewed by non expert readers. The least experienced reader benefitted the most from the additional CTP and 4D-CTA (Table 2).

CTP can provide additional data on stroke patients in the acute setting [14–16] and is used routinely in acute stroke imaging in modern clinics globally despite being subject to heavy debate and often. Clinics who are already performing CTP don’t need to perform scanning, by adding 4D-CTA to the post-processing protocol, avoiding the need for additional radiation dose or contrast agent. Quality of 4D-CTA might fluctuate in patients with poor cardiac output, due to more difficult scan timing. In stroke patients movement artifacts can be an issue. However compared to CTP movement artifacts in the 4D-CTA are less of a problem. CTA is also subject to movement artifacts and dependent on cardiac output. Venous contamination is a well-known pitfall in stroke imaging where venous structures can be mistaken for patent arterial vessels, leading to false-negative findings. Compared with CTA a major benefit of 4D-CTA is absence of venous filling in the early phases of the scan, allowing better differentiation between arteries and venes. A hyper dens media sign on CTA, mimicking a patent vessel, can also lead to false negative findings. [17]

Perfusion imaging in this study showed no improvement in accuracy: although it resulted in a slight increase in sensitivity, it led to a decrease in specificity in some readers. This is caused by false positive readings in patients with initially declared patent vessels after finding a large core and/or penumbra on CTP this was changed to occlusions. In clinical practice false positive findings can lead to unnecessary interventional procedures.

No standardized viewing of the regular CTA was requested, the readers were free to use MIP, volume rendering and/ or bone-removal reflecting daily practice. Observations show that some reviewers prefer coronal MIP/MPR where others prefer source data. No clear overall preference was seen. Optimal viewing post-processing for CTA remains to be published.

In our clinic we observe that even the most experienced neuro- and interventional radiologist readers use the 4D-CTA to fast-determine intra-cranial occlusions, flow dynamics and cloth burden. As previously published 4D-CTA gives more accurate information on cloth burden and collateral supply to the infarcted area compared to regular CTA, thereby providing more detailed information to the interventional radiologist and possibly important prognostic data on patient outcome. [9] The ability to differentiate a true tandem occlusion (intracranial large vessel occlusion in combination with extra-cranial Internal Carotid Artery (ICA) occlusion or stenosis) from pseudo tandem occlusion (intra cranial occlusion with patent extra cranial ICA) is a known benefit of 4D-CTA, with major prognostic and therapeutic implications. [18]

**Fig 4:**
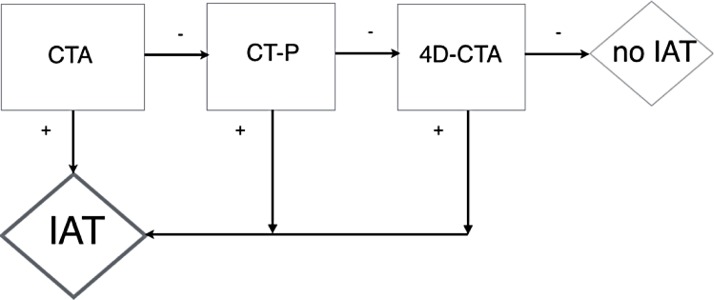
Patient selection protocol.

4D-CTA takes 4 to 11 minutes extra time to process and review as shown in our study. To avoid patient delay in the clinical setting first regular CTA should be reviewed. When an occlusion is present the patient is then directly transferred to the angio-suite for IAT, before further reviewing the CTP and/or 4D-CTA. In case of patent declared vessels on the initial CTA, further assessment of CTP and 4D-CTA can improve diagnostic accuracy, without causing patient delay. In this way there is optimal benefit for all patients; A minutes long delay in eligible patients with apparent occlusion on the CTA is prevented by direct transfer to the angiosuite, while patients with initial false negative declared patent vessels still benefit from improved accuracy of the 4D-CTA, when missed occlusion is detected after viewing the 4D-CTA. Our patient selection protocol is demonstrated in Fig 4.

It is expected that further advantages in post processing algorithms and software applications will shorten the time needed to calculate and post process the images in 4D-CTA.

The small number of patients included in our pilot study gives limited statistical power, tough clearly displays beneficial results for 4D-CTA, indicating high potential for further research. Methodological strengths of our study were the strict, consecutive and unselected inclusion (according MR CLEAN inclusion) and blinded reviewing.[4] Furthermore, normal clinical practice was simulated by allowing reviewers to post-process the images manually while time metrics were recorded.

Future research is aimed at 4D-CTA as first step imaging analysis, were 4D-CTA could be a reliable predictor of vessel patency. In clinical practice this is only feasible if reconstruction and post processing time of the 4D-CTA are optimized.

## Conclusion

Our pilot study shows that 4D-CTA derived from CTP is a relatively fast and reliable additional technique to the standard imaging protocol of NECT, CTA and CTP in patients with acute ischemic stroke, when reviewed by non-experts such as radiologists in training. It shows an increase in diagnostic certainty in clinical decision-making and a tendency to improve diagnostic accuracy, while causing only minor delay in image reviewing in patients with patent vessels on CTA. Therefor 4D-CTA seems able to optimize patient selection for IAT. Given the small number of patients, confirmation of larger studies in the future is required.

## Abbreviations

IAT: Intra-arterial therapy
NECT: Non Enhanced CT
CT: Computed Tomography
CTA: Computed Tomography Angiography
DE-CTA: Dual Energy CTA
CTP: CT Perfusion
4D-CTA: 4Dimensional CTA / Time resolved CTA
